# Identification of Genes from the Fungal Pathogen *Cryptococcus neoformans* Related to Transmigration into the Central Nervous System

**DOI:** 10.1371/journal.pone.0045083

**Published:** 2012-09-20

**Authors:** Hsiang-Kuang Tseng, Chang-Pan Liu, Michael S. Price, Ambrose Y. Jong, Jui-Chih Chang, Dena L. Toffaletti, Marisol Betancourt-Quiroz, Aubrey E. Frazzitta, Wen-Long Cho, John R. Perfect

**Affiliations:** 1 Institute of Clinical Medicine, National Yang-Ming University, Taipei, Taiwan; 2 Division of Infectious Diseases, Department of Internal Medicine, Mackay Memorial Hospital, Taipei, Taiwan; 3 Division of Infectious Diseases, Department of Medicine, Duke University Medical Center, Durham, NC, United States of America; 4 Division of Hematology-Oncology, The Saban Research Institute, Children’s Hospital Los Angeles, Department of Pediatrics, Keck School of Medicine, University of Southern California, Los Angeles, California, United States of America; 5 Division of Thoracic and Cardiovascular Surgery, Buddhist Tzu Chi General Hospital, Hualien, Taiwan; 6 Department of Medicine, Mackay Medical College, New Taipei, Taiwan; University of Minnesota, United States of America

## Abstract

**Background:**

A mouse brain transmigration assessment (MBTA) was created to investigate the central nervous system (CNS) pathogenesis of cryptococcal meningoencephalitis.

**Methodology/Principal Findings:**

Two cryptococcal mutants were identified from a pool of 109 pre-selected mutants that were signature-tagged with the nourseothricin acetyltransferase (NAT) resistance cassette. These two mutants displayed abnormal transmigration into the central nervous system. One mutant displaying decreased transmigration contains a null mutation in the putative *FNX1* gene, whereas the other mutant possessing a null mutation in the putative *RUB1* gene exhibited increased transmigration into the brain. Two macrophage adhesion-defective mutants in the pool, 12F1 and 3C9, showed reduced phagocytosis by macrophages, but displayed no defects in CNS entry suggesting that transit within macrophages (the “Trojan horse” model of CNS entry) is not the primary mechanism for *C. neoformans* migration into the CNS in this MBTA.

**Conclusions/Significance:**

This research design provides a new strategy for genetic impact studies on how *Cryptococcus* passes through the blood-brain barrier (BBB), and the specific isolated mutants in this assay support a transcellular mechanism of CNS entry.

## Introduction

*Cryptococcus neoformans* is a haploid yeast, and its natural history of growth and disease production in human hosts are well known. Infection generally presents in body sites such as the lung, bloodstream, and specifically the CNS, resulting in life-threatening cryptococcal meningoencephalitis [1]. Cryptococcal meningoencephalitis is universally fatal if untreated; even with treatment and advanced medical care, 20–25% mortality may be observed [2]. It is an AIDS-defining disease that has become the fourth leading cause of mortality due to infection diseases in sub-Saharan Africa with an estimated one million cases and over 600,000 deaths per year [3]. Obviously, the current global burden of HIV-related cryptococcosis is substantial [4].

Fungemia likely influences the neurotropism observed in disseminated cryptococcosis. Since the direct movement of this yeast from the lungs to the brain is not pathophysiologically feasible, the bloodstream is the likely conduit for cryptococcal dissemination from its initial lung infection to crossing of the blood-brain barrier (BBB) [5]. Using an experimental meningoencephalitis model, circulating *C. neoformans* yeast cells have been shown to invade brain endothelium under shear stress following a capillary microembolic event [6]. In addition to mechanical trapping within the blood vessels, yeast cells must also remain viable in order to cross through the brain microvasculature [6], [7]. Using an intravenous murine model, viable yeasts have been identified in brain homogenates as early as five minutes after intravenous inoculation [7]. After rapid localization to the brain, the viable fungal burden decreases slightly and then stabilizes for the next 24 hrs. After this initial period of adjustment, the fungal burden in the brain increases rapidly, demonstrating intracranial yeast growth and disease [7].

Since biolistic gene deletion methods for *C. neoformans* were introduced in 1993 [8], many targeted gene deletion mutants have been reported in the literature; screening of these mutants in animal models has identified and validated genes essential for cryptococcal pathogenesis [9]. Furthermore, signature-tagged mutagenesis (STM) is a powerful negative selection screening technique for globally identifying pathogenesis-related genes in host models [10]. With the release of the *C. neoformans* genome sequence [11], Liu et al. constructed a gene deletion library containing over 1200 mutants, representing approximately one-fifth of the genes in *C. neoformans* [12]. Thus, global strategies for the screening and identification of unbiased virulence determinant mutants of *C. neoformans* can be performed [13].

**Figure 1:**
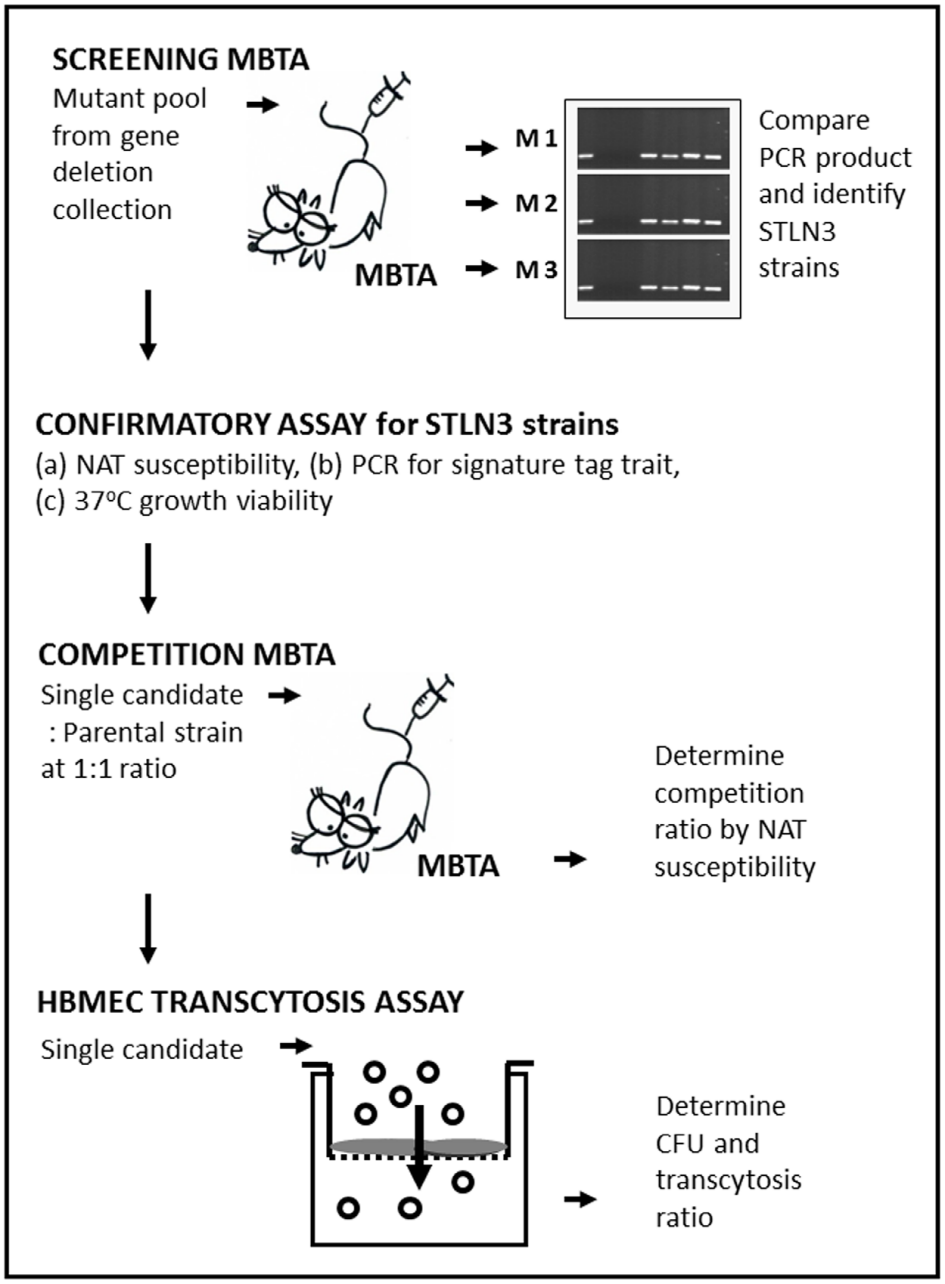
The overall chart of strategy. The screen and identification of *Cryptococcus neoformans* mutants for CNS transmigration phenotype in this study through MBTA and HBMEC are shown. Abbreviation: MBTA: Mouse brain transmigration assay, HBMEC: human brain microvascular endothelial cells, NAT: nourseothricin acetyltransferase, M1: first mouse, M2: second mouse, M3: third mouse, STLN3: signature tag loss number is three in all three mice.

We used this gene deletion library to design an innovative screening strategy for identifying specific genes required for efficient *C. neoformans* trafficking into the CNS (**Figure 1**): (1) screening of mutants using the *in vivo* mouse brain transmigration assay (MBTA) to identify candidates from the original mutant library; (2) individually confirm the phenotype of these mutant candidates *in vitro*; (3) confirmation of MBTA results *in vivo* using a 1∶1 ratio of each mutant with the parental strain; (4) confirmation of the microvascular entrapment phenomenon *in vivo* [6] using *in vitro* transcytosis assays using human brain microvascular endothelial cells (HBMECs) with selected mutants to mechanistically confirm that these mutants exhibit altered endothelial transmigration. With this strategy, we identified several cryptococcal mutants that are altered in HBMEC transcytosis compared to the parental strain. Moreover, we have examined these BBB-related mutants for binding and translocation through HBMECs. This mutant identification process allows modeling of the mechanism(s) of BBB crossing by *C. neoformans* [7], [14]–[17].

Our present understanding of pathophysiology supports three possible routes of BBB crossing for *C. neoformans*: (1) transcellular migration through HBMEC with identification of specific ligand-receptor interactions [14], [15], [18]; (2) paracellular migration between the endothelial cells after mechanical or biochemical disruption of the BBB [7], [14], [16]; and (3) “Trojan horse” model in which yeast cells cross BBB inside a host monocyte [17]. For example, *CPS1*, encoding hyaluronic acid synthase, is important for transcellular migration of *C. neoformans* across a HBMEC monolayer [19], [20]. However, an *ure1*Δ (urease) mutant appears to cross the BBB via paracellular migration [16]. Therefore, we used both *cps1*Δ and *ure1*Δ mutants to discriminate between the transcellular and paracellular routes of BBB transmigration in this MBTA. Furthermore, we assessed the potential macrophage-mediated transmigration using macrophage adhesion-defective mutants. We predict that these mutants would not be able to travel inside mononuclear cells *in vivo*. Thus, if yeast cells in this MBTA had improved transit across the BBB inside host cells, these adhesion-deficient mutants would be predicted to have reduced BBB transmigration efficiency.

Using our MBTA and *in vitro* HBMEC, we identified two novel mutants with significantly altered BBB passage. The study demonstrates the potential influence of genetic control for efficient yeast transmigration into CNS and begins to discriminate among several competing mechanisms for efficient yeast CNS entry.

## Results

### Generation and Screening of Customized Pools of Cryptococcal Mutants

**Figure 2:**
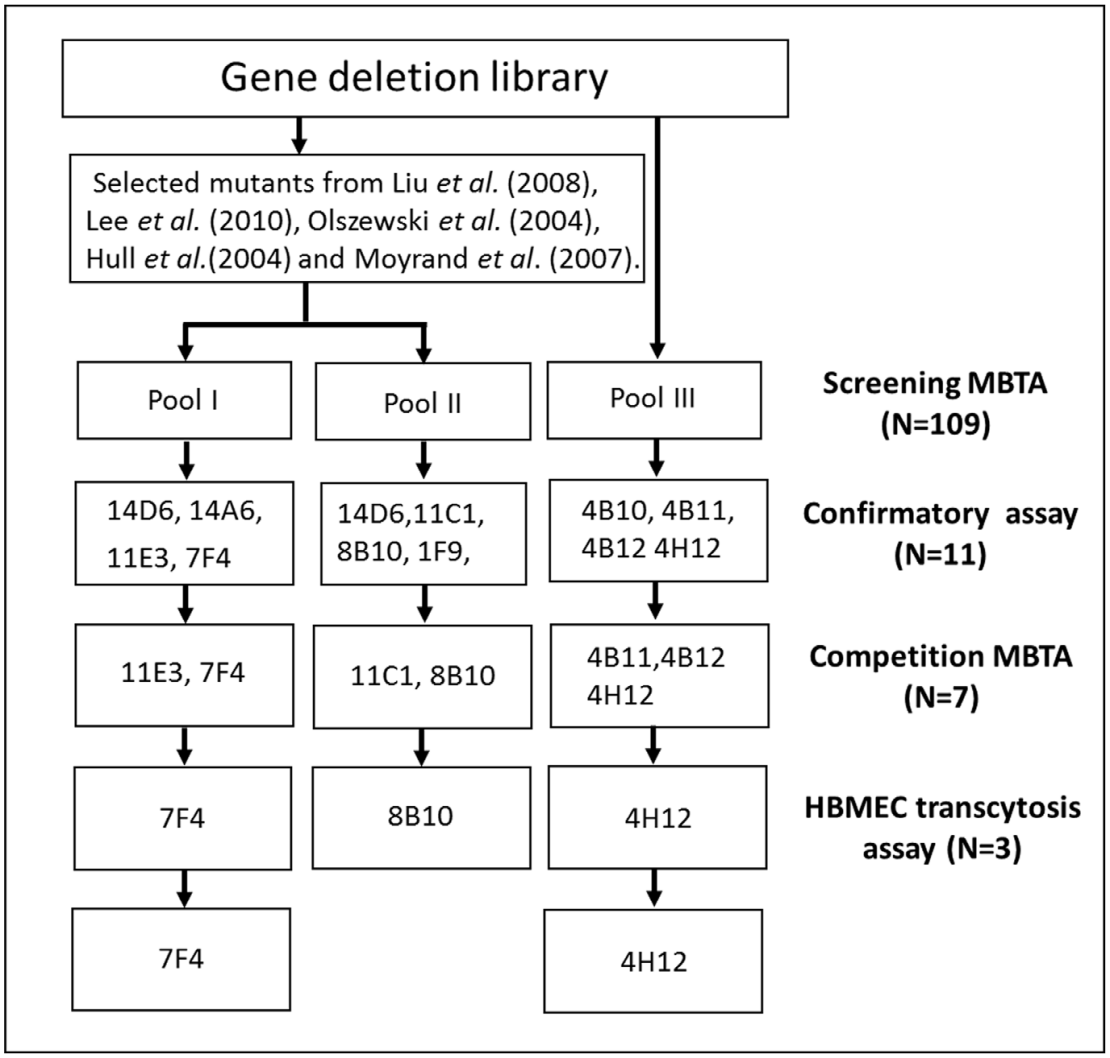
The flow chart of 109 cryptococcal mutants in three pools tested in this study. The 7F4 clone and 4H12 clone are identified as BBB transmigration related mutants.

In order to efficiently evaluate and verify the MBTA model, we pooled selected mutants involved in CNS pathology of *C. neoformans* **(** **Figure 2**). A general hypothesis in these studies was that mutants that are defective in lung infectivity [12] or CNS survival [21] may also be affected in CNS entry. Therefore, we generated two customized pools of mutants (Pool I and II) for our screen **(** **Table 1**), utilizing the 48 unique signature tags in the published gene deletion library and rearranging them in one 96-well tissue culture plate. Briefly, Liu and colleagues [12] screened the entire library for *in vivo* proliferation in murine lung tissue and *in vitro* for three well-recognized virulence attributes: capsule formation, melanization, and growth at 37°C. We selected 65 of the 82 novel capsule, melanin and “lung” infectivity mutants identified in their study. These 65 strains included four of the 13 mutants identified in the study of Lee and colleagues [21] for survival defects in growth media, saline, and human cerebrospinal fluid (CSF): 1A10 (*ena1*Δ), 7F4 (*rub1*Δ), 8F3 (*hrd1*Δ), and 12F1 (*vam6*Δ). Additional mutants were selected based on prior studies: 14A4 (*pik1*Δ) and 12G8 (*cps1*Δ) [21]; 1F12 (*cap64*Δ), 2F6 (*ugt1*Δ), 3B12 (*uge4*Δ) [22]; 14D6 (*ure1*Δ) [16]; along with two deletion library controls, 9H6 (*sxi1*Δ) and 12H12(*sxi1*Δ) [23]. Mutant strains 12G8 and 14D6 were assigned to both customized pools and were used to compare their established brain transmigration defects (transcellular and paracellular, respectively) with the pooled mutants. Also, their specific CNS transmigration mechanisms were tested individually in our system. Therefore, Pool I and Pool II contained 35 and 40 strains, respectively. Additionally, the 37 mutants in Pool III included 4B10 (*nsr1*Δ) (a CSF survival defect mutant in Lee’s study [21]) are a randomly chosen set of mutants from the original gene deletion library **(** **Table 1**). Among 109 mutants screened in this study, 1A10 (*ena1*Δ), 7F4 (*rub1*Δ), 8F3 (*hrd1*Δ), 12F1 (*vam6*Δ), 14A4 (*pik1*Δ), 12G8 (*cps1*Δ) and 4B10 (*nsr1*Δ) were identified as CSF survival-defective mutants in prior work [21].

**Table 1:** Summary of 109 *Cryptococcus* genes tested in this study.

Pool I^a^	1	2	3	4	5	6
**A**	*–*	*LIV1*	*RPD304*	*PIK1*	*–*	*–*
**B**	*SNT1*	*GAT201*	*–*	*YCK2*	*LIV7*	*–*
**C**	*HSV2*	*–*	*MLN2*	*–*	*UBP14*	*LIV9*
**D**	*PAN1*	*FYV10*	*LIV6*	*CUL3*	*CSN7*	*Urease*
**E**	*–*	*–*	*YKU80*	*RHO104*	*LIV13*	*CPL1*
**F**	*VAM6*	*–*	*HRD1*	*RUB1 (RPS3102)*	*LIV5*	*UGT1*
**G**	*LIV11*	*CPS1*	*ZAP103*	*–*	*–*	*MLN3*
**H**	*LIV10*	*–*	*CSN1*	*CDC2801*	*JJJ1*	*SXI1*
**Pool II** b	***7***	***8***	***9***	***10***	***11***	***12***
**A**	*–*	*HOS2*	*SSN801*	*ENA1*	*SNF102*	*KSP1*
**B**	*–*	*–*	*–*	*SET302*	*NHP6B02*	*UGE4*
**C**	*LIV2*	*NHP6B01*	*RTF1*	*UBA4*	*MLN1*	*MLN4*
**D**	*–*	*HIR1*	*RAD54*	*LSB1*	*MBP102*	*Urease*
**E**	*HST302*	*–*	*HRK1*	*SET101*	*RIM20*	*CSN4*
**F**	*RGD1*	*–*	*RINT1*	*HSE102*	*SET202*	*CAP64*
**G**	*LIV14*	*CPS1*	*RIM101*	*RAD502*	*LIV4*	*LIV15*
**H**	*LIV12*	*LIV16*	*–*	*CHO2*	*UBC8*	*SXI1*
**Pool III** c	***7***	***8***	***9***	***10***	***11***	***12***
**A**	*UBP801*	*ORF*	*ORF*	*MLH1*	*ORF*	*PSF3*
**B**	*ORF*	*ORF*	*NTH2*	*NSR1*	*CMK101*	*ARR3*
**C**	*–*	*Replaced*	*–*	*MSH3*	*–*	*CSF1*
**D**	*KIP101*	*SKN703*	*–*	*ORF*	*CDC10*	*YOR291W*
**E**	*ORF*	*ORF*	*–*	*–*	*KIP2*	*Replaced*
**F**	*ORF*	*Replaced*	*UBC1302*	*MSH201*	*TKL103*	*MGS1*
**G**	*ORF*	*SKN702*	*UME6*	*ORF*	*CRN102*	*CNH1670*
**H**	*HSL102*	*–*	*BAT2*	*ORF*	*–*	*FXN1 (YMR088C02)*

a Pool I included 35 genes;

b Pool II: 40 genes;

c Pool III: 37 genes. “–“: blank plate addresses. “Replaced”: mutants in plate addresses were replaced. *CPS1*, *Urease* and *SXI1*were assigned to both customized pools (Pool I and Pool II).

### Identification of Candidate Mutants Using the MBTA Model

Each mutant pool was separately assessed using three mice. Mice were sacrificed and perfused with saline to avoid contamination of brain tissues by circulating yeasts within the vasculature. Negative selection of candidate mutants from the MBTA screen was performed by amplifying signature tags via PCR. Therefore, if the amplicon was lost in all three mice per group, the signature tag loss number (STLN) was 3, and those candidates were designated as STLN3 strains in this study. We selected 11 STLN3 mutant candidates based on their complete absence from brain tissue 14D6, 14A4, 11E3, 7F4 from Pool I; 14D6, 1F9, 11C1, 8B10 from Pool II; 4B10, 4B11, 4B12, 4H12 from Pool III **(** **Figure 2**).

### Confirmation of STLN3 Strains via NAT Susceptibility, Signature Tag PCR, and 37°C Growth

To exclude possible contaminants, STLN3 strains were grown in the presence of nourseothricin (NAT) to confirm the activity of the inserted NAT resistance (NAT^R^) marker. Each of the mutants derived from the mutant library possesses unique signature tag sequences, and these tags were used to identify each strain by PCR. However, three of the library mutants were unable to be identified by PCR amplification of the signature tags: 14D6, 1F9 or 14A4. We excluded two mutants from further analysis but kept 14D6 because of our ability to identify it as urease-negative using a colorimetric assay [24].

**Figure 3:**
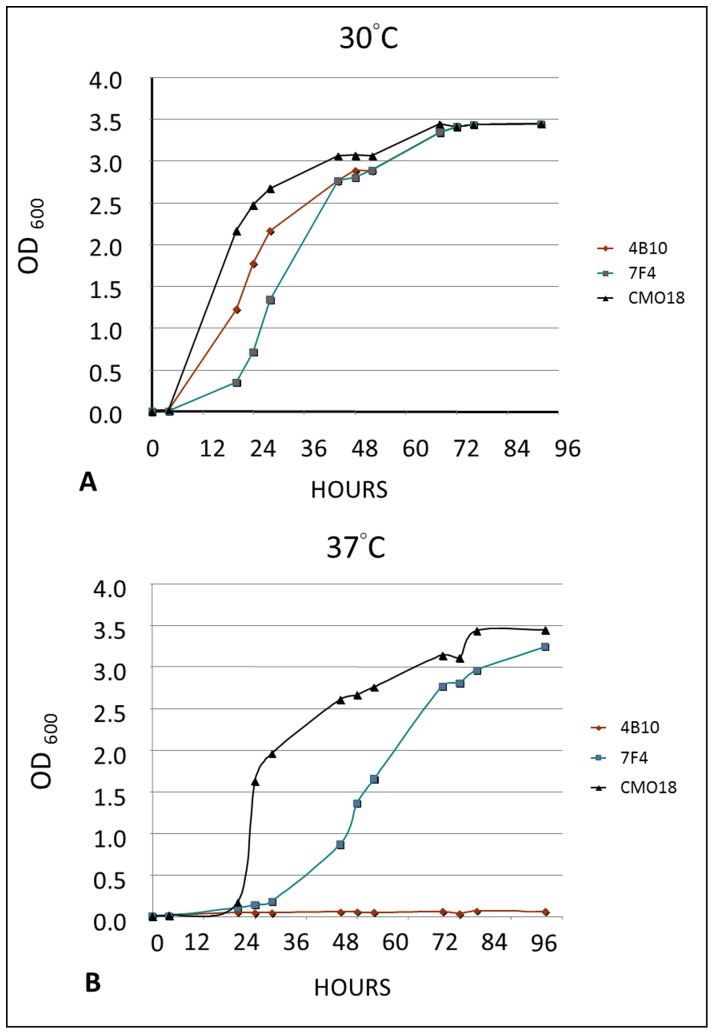
30°C and 37°C growth curve. At 30°C, 4B10 and 7F4 in shaking incubator showed reduced growth compared to parental strain. At 37°C, 4B10 exhibited significant growth arrest whereas 7F4 exhibited a much slower growth than parental strain but reached a growth plateau after 72 hours.

Viability of *Cryptococcus* yeast cells in host tissue (at 37°C) is essential for traversal of the BBB [6], [7]. Temperature sensitivity at 37°C was initially evaluated by directly plating individual STLN 3 mutants on culture plates and incubating them at 37°C, 5% CO_2_ for 2 days. We identified two mutants, 4B10 and 7F4, that showed reduced growth at 37°C. To separate thermosensitivity from overall slower growth, we further evaluated these two mutants in growth curve assays at 30°C and 37°C (**Figure 3**). Mutant 4B10 exhibited slow growth at 30°C but a significant growth arrest at 37°C, whereas 7F4 exhibited slower growth than the parental strain but was not completely restricted at 30°C or 37°C. Therefore, we excluded 4B10 from further analysis, since its thermosensitivity phenotype may contribute to its exclusion from brain tissue, yet we kept 7F4, which needed a longer period of time (2–3 days at 30°C, and 3–4 days 37°C) to reach stationary phase.

### Competitive MBTA Using the Parental Strain CMO18

We performed a MBTA comparing the individual STLN 3 mutants to the parental strain CMO18 to reconfirm differences observed in the first MBTA screen, to remove any potential group effect among mutants in the original MBTA screen, and to precisely measure differences in yeast transmigration efficiency. NAT susceptibility was also used as a tool to differentiate mutant strain colonies (NAT resistant) from parental strain CMO18 colonies (NAT susceptible) in our experimental MBTA model. Based on this assay, we excluded mutant strains 4B12, 4B11, 11E3, and 11C1 due to lack of significant difference in BBB traversal between each mutant and the parental strain (**Table 2**). However, we did identify 2 mutants (4H12 and 8B10) showing significantly lower fungal burden (competition ratio <1.00) than the parental strain by using the direct competitive MBTA. Since mutant 7F4 is a slow grower yet is viable at 37°C (**Figure 3**), we extended the incubation time of colonies from 2 days to 4 days to obtain visible colonies of 7F4 on plates and obtain accurate measurement of 7F4 colony forming units (CFU). Surprisingly, mutant 7F4 showed significantly higher fungal burden (competition ratio = 2.72±0.44) compared to the parental strain (**Table 2**).

**Table 2:** The competition ratio of seven STLN3 strains by MBTA.

Pool	Candidate	Gene Name	Parental Strain	Ratio in Inoculum	Ratio in Brain(Mean ± SD)	*P* value
**Pool I**	11E3	*YKU 80*	CMO18	1.00	1.04±0.17	0.849
**Pool I**	7F4	*RUB1*	CMO18	1.00	2.72±0.44	0.017
**Pool II**	11C1	*LIV 2*	CMO18	1.00	0.97±0.12	0.827
**Pool II**	8B10	*SET 302*	CMO18	1.00	0.74±0.07	0.037
**Pool III**	4B12	*ARR3*	CMO18	1.00	0.72±0.11	0.079
**Pool III**	4B11	*CMK101*	CMO18	1.00	0.70±0.22	0.264
**Pool III**	4H12	*FXN1*	CMO18	1.00	0.78±0.06	0.017

Abbreviation: MBTA: Mouse brain transmigration assay, STLN3: signature tag loss number is three in all three mice.

### Confirmation of BBB Traversal Mutants *in vitro*

**Figure 4:**
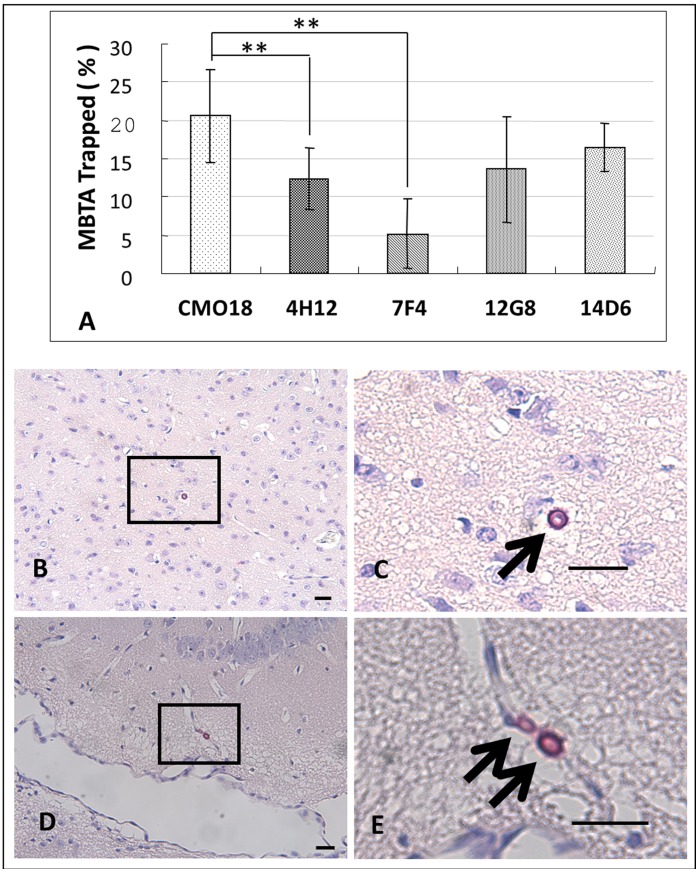
Entrapment MBTA. (A) Each yeast strain demonstrates different entrapped yeast percentage within vascular compartment. (B & C) *Cryptococcus* located in brain parenchymal (outside of microvessel); and (D & E) *Cryptococcus* trapped within the brain microvasculature (inside of microvessel). Yeast cells are stained with mucicarmine (arrow bar: 10 µm). *P* value is determined by Student’s t test.

According to previous reports using the murine model of disseminated meningoencephalitis to study the early events associated with BBB crossing [6], [7], there remained a percentage of *Cryptococcus* cells entrapped in microvasculature of brain. Similarly, our results of the MBTA entrapment assay showed that microvascular entrapped percentages varied among different strains of *Cryptococcus* after 24 hours although we performed circulatory perfusion to avoid contamination of brain tissue by cryptococcal yeast cells in the blood (**Figure 4**). According to our data most of the yeasts (79%–95%) were found scattered within the brain parenchyma compartment (**Figure 4B, 4C** **)** but occasional yeasts (5%–21%) were found directly in small blood vessels of the brain’s vascular compartment, as trapped within microvessels (**Figure 4D, 4E** **)**. In fact, compared to CMO18, the abnormal transmigration candidates 4H12 and 7F4 possessed a significantly lower ratio of entrapped yeasts compared to CMO18 in the MBTA model (**Figure 4A**). This finding indicated that the transmigration-related candidates identified in the MBTA competition assay may be affected by a microvascular trapping phenomenon.

**Figure 5:**
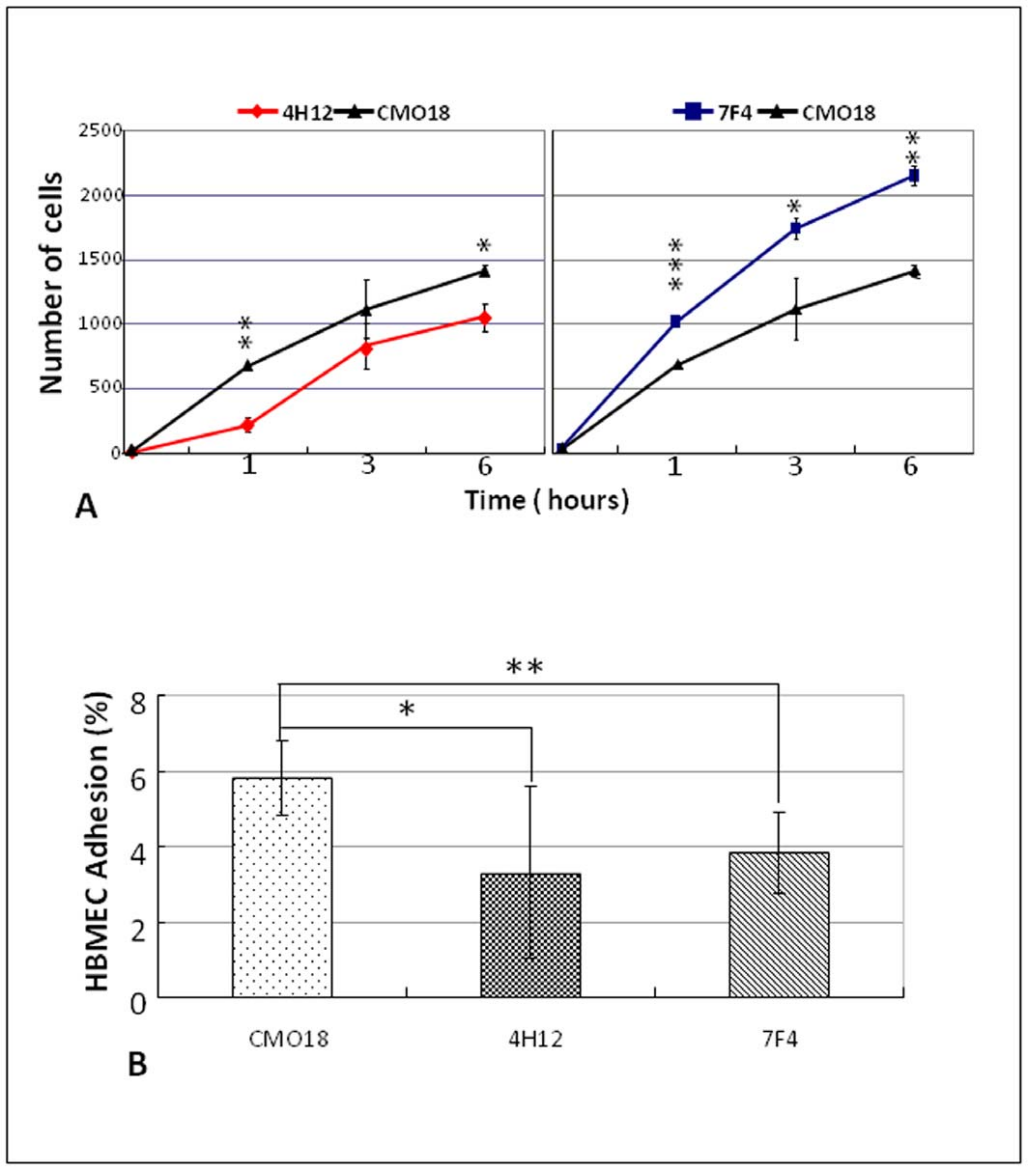
Transcytosis and adhesion assays of HBMEC. (A) HBMEC transcytosis assay. Mechanistic confirmatory results of mutants 4H12 and 7F4. Yeast cell numbers of *C. neoformans* mutants and parental strain CMO18 in lower chamber of transcytosis assay at different time point (1 hr, 3 hrs, and 6 hrs) are demonstrated. Compared to parental strain CMO18, 4H12 is significant lower ability and 7F4 is significant higher ability in transcytosis. *P* value is determined by Student’s t test. (B) HBMEC adhesion assay. Campared with parental strain CMO18, the 4H12 and 7F4 strains exhibit lower adhesion percentage to the HBMEC than the parental strain. *P* value is determined by Student’s t test.

Next, we examined whether a specific *in vitro* assay would help us confirm our *in vivo* observations and attempt to understand specific mechanism(s) for crossing. We designed a HBMEC transcytosis assay to measure the ability of the *C. neoformans* mutants to traverse the microvasculature in comparison to the parental strain over time. We identified mutant 4H12 showed significantly reduced transcytosis compared to the parental strain at 1 hr and 6 hr (Figure 5A). Consistent with the MBTA model *in vivo*, mutant 7F4 showed significantly enhanced transcytosis compared to the parental strain in the HBMEC model *in vitro* at 1 hr, 3 hr, and 6 hr (Figure 5A). Before yeast transmigrate across the BBB, an important step for *C. neoformans* is to attach to the brain endothelium cells actively or passively. Therefore, the active binding ability of MBTA candidates was also evaluated using a HBMEC binding assay. Compared to the parental strain CMO18, 4H12 showed 1.7-fold lower adhesion (**Figure 5B**). Also, the 7F4 strain also exhibited 1.5-fold lower adhesion in the HBMEC assay compared to the parental strain (**Figure 5B**). While 4H12 showed both decreased adherence and movement across the HBMEC, 7F4 showed decreased adherence to the HBMEC**,** but increased transmigration across the HBMEC monolayer *in vitro* **(** **Figure 5** **)**. Although generally adhesion and transcytosis are proportional we suspect 7F4 may have traversed faster across the membrane in the assay than the adhesion was measured and demonstrates the dynamic complexity of yeast migration across membranes.

**Figure 6:**
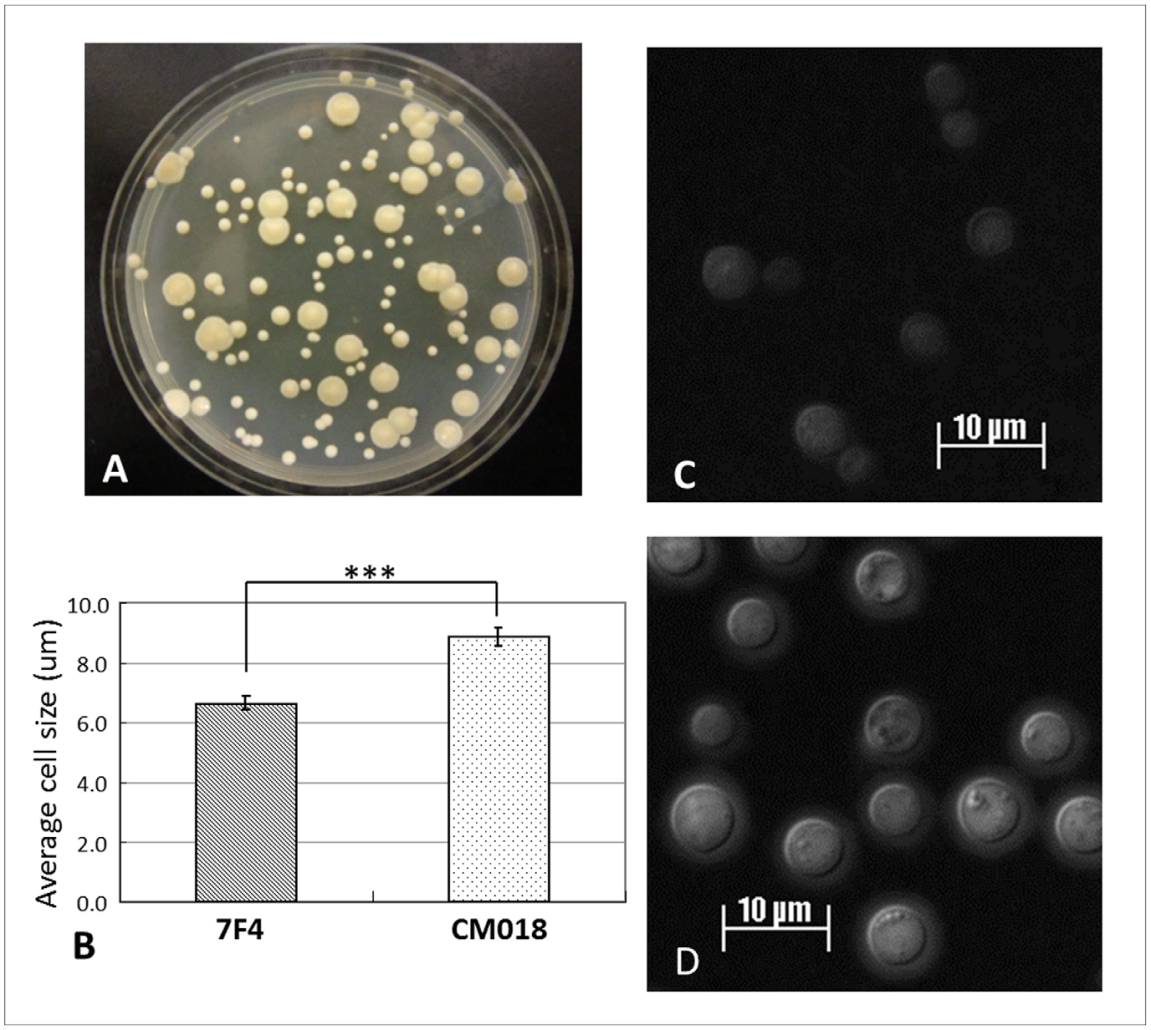
The “petite” cells of 7F4 in comparison with parental strain CMO18. (**A**) The small sizes of 7F4 colonies compared to that of CMO18 in SDA/c culture plate after 4-day growth at 30°C. (**B**) The unit on y axis is µm, on a 200% adobe photoshop picture, from a 60x Zeiss microscope image. Adobe Photoshop measures in relative units. Total 100 cells examined per strain and total 5 images per strain (differences in diameters *P*<0.0001). *P* value is determined by Student’s t test. (**C**) 7F4 (*rub1*Δ) and (**D**) parental strain CM018 with an India Ink stain under oil immersion magnification after 48 hours in Dulbecco's Modified Eagle Medium (DMEM) at 30°C shaking incubator.

One interesting morphological feature of the 7F4 is its smaller size of colony and yeast cell than the parental strain CMO18 (**Figure 6A**). Physically, 7F4 cell has a much smaller cell diameter (mean 6.6±0.2 mm) than the parental strain CM018 (8.8±0.3 mm) (*P*<0.0001) **(** **Figure 6** **B, C, D)**. The small (petite) cells of 7F4 which appear to increase BBB crossing are in contrast to cryptococcal “titan cells” (large cells) that possess reduced ability to penetrate BBB [25]. Thus, yeast cell morphology may significantly affect host cell interactions and pathogenicity in relationship to brain entry [26]. In fact, despite reduced adhesion to HBMECs, the small size may have helped 7F4 to pass efficiently across the BBB.

### Competitive MBTA for Testing the Role of Transcellular and Paracellular Entry in CNS Dissemination of *C. neoformans*

In order to confirm potential mechanisms for our STLN3 mutants in MBTA system we examined a known transcytosis mutant (*cps1*Δ) as a control for the MBTA. In our system, mutant 12G8 (*cps1*Δ) did exhibit significantly reduced transmigration in mice brain homogenates (competition ratio = 0.66±0.03, *P = *0.0004) by competitive MBTA. The data is consistent with the previous reports that *CPS1* gene is important factor for traversal of *Cryptococcus* into brain via a transcellular route [19], [20]. With mutant 14D6 (urease mutant) we had the opportunity to examine MBTA’s ability to detect paracellular entry. We tested 14D6 in the competitive MBTA and the competitive ratio was 1.51±0.22 (*P = *0.078). Thus, the urease mutant 14D6 did not have a significant CNS transmigration defect compared to the parental strain in this MBTA. Taken together, results from 12G8 and 14D6 support a transcellular mechanism of CNS entry as the primary mechanistic role in our system. However, our result doesn’t exclude the potential importance of a urease dependent paracellulinto brain via a transcellular route

ar mechanism in models examining brain invasion for longer periods [6]. Furthermore, urease activity is clearly not necessary for the yeast’s ability to cross the BBB in humans since there are several case reports of urease negative strains of *C. neoformans* that have been isolated from CNS infections in patients with AIDS [27], [28]. On the other hand, it likely still contributes to the BBB migration of yeast cells as previously described [16].

### Evaluation of the “Trojan Horse” Entry Route in the MBTA

In an attempt to appreciate the impact of yeast cells being carried into CNS inside host cells with our MBTA, we identified mutants which would not survive or attach to professional phagocytes and thus would likely be free at all times in the blood stream. According to the PCR results of screening experiments by mouse macrophage phagocytosis/survival assay (MMPA) using a macrophage-like cell line, we selected 5 mutants that yielded missing signature tags in the lysis (L) pools from the macrophage survival study: 12D10 (*lsb1*Δ), 5E4 (*rho104*Δ), 5A12 (*ksp1*Δ), 12F1 (*vam6*Δ), and 3C9 (*rtf*Δ). We then tested these five mutants in the adhesion study of the MMPA at 1 hour and the adhesion percentage with macrophage were 88.77±2.11% for 12D10 (*P* = 0.366), 90.49±4.39% for 5E4 (*P* = 0.478), and 95.94±0.31% for 5A12 (*P* = 0.152). On the other hand, we identified a significant low adhesion percentage with the macrophage-like J774.A1 cell line compared to the parental strain in 12F1 (9.29±8.81%, *P* = 0.045) and 3C9 (15.26±4.74%, *P* = 0.032).

The gene target disrupted in mutant strain 12F1 (CNAG_05395; http://www.broadinstitute.org/annotation/genome/cryptococcus_neoformans/MultiHome.html) encodes a homolog of the RabGEF (Guanyl-nucleotide Exchange Factor) *VAM6* in *Saccharomyces cerevisiae*. There are at least nine *VAM* genes grouped into two classes according to the mutant phenotypes in *S. cerevisiae* [29]. The class II VAM mutants (*VAM2, VAM3, VAM4, VAM6*, and *VAM7*) include mutants with small vesicles and mature forms of the vacuolar proteins and do not show any obvious growth defects in elevated CaC1_2_ or at 37°C [29].The gene disrupted in mutant strain 3C9 (CNAG_06648) encodes a homolog of *RTF1*, a RNA polymerase II Transcription Elongation Factor protein in *S. cerevisiae*. Interaction of the TATA box-binding protein (TBP) with promoters of RNA polymerase II-transcribed genes is an early and essential step in mRNA synthesis. Rtf1p either directly or indirectly regulates the DNA binding properties of TBP and, consequently, the relative activities of different TATA elements *in vivo* [30].

Since both 12F1 and 3C9 appear to be macrophage adhesion defective mutants, we next indirectly evaluated the potential impact of the “Trojan horse” route for BBB traversal in this MBTA. These mutants would not easily be taken up by phagocytic cells within the bloodstream and thus unlikely to be able to cross BBB inside host cells. The results of competition ratios of 12F1 (1.48±0.25, *P* = 0.130) and 3C9 (1.33±0.14, *P* = 0.082) were higher than 1.00 in the individual competitive MBTA. Despite the reduced ability of these strains to bind professional phagocytes, these mutants traveled across the BBB very efficiently in our MBTA and were similar to strains which could effectively bind to macrophages. Therefore, the “Trojan horse” route of entry by *C. neoformans* is not likely to be well in our MBTA. However, our studies do not exclude the existence of the “Trojan horse” method in cryptococcal traversal BBB under other circumstances such as when yeast cells are traveling to the brain from an established lung infection [17].

## Discussion

### The Usefulness of MBTA

Since cryptococcal CNS pathogenesis is a complex phenomenon involving increasing numbers of cryptococcal virulence genes that have been identified for the virulence composite, we realized that a combination of virulence genes and their controlling mechanisms rather than a single target or pathway are likely involved in the multiple steps of cryptococcal dissemination to the CNS and particularly, BBB transmigration. Thus, in this manuscript we discuss a well-defined strategy to screen many genes using a MBTA model. This MBTA model represents an initial pilot design to screen for mutants with CNS transmigration alternation. We identified mutants with proven infectivity defects in two customized pools (Pool I and II) and one chosen pool (Pool III) from the available gene-deletion collection **(** **Figure 2** **)** and demonstrated that high-throughput screening of an entire mutant library is possible.

Our initial results with the lung infectivity mutants found in Liu’s studies [12] identified only one (7F4) with a CNS transmigration alteration out of 65 lung infectivity mutants; therefore the concordant identification rate was only 1.5% (1/65) from Liu’s collection. Thus, infection sites clearly place differ demands on the yeast. Second, among seven of the thirteen mutants with CSF survival defects found in Lee’s studies [21], two candidates (7F4 and 12G8) were shown to exhibit abnormal transmigration into the CNS in our system. Since this corresponding identification rate was 28.6%, (2/7), we may identify one to two more candidates related to transmigration into CNS among the six remaining CSF survival defective mutants. Clearly, genetic control of brain infectivity and optimal survival once in this tissue/fluid are not always concordant.

There were 37 mutants in the randomly chosen Pool III that was originally designed as a control pool for comparison with the customized Pools I and II. We identified one mutant (4H12) associated with abnormal BBB transmigration from this pool; hence, the positive identification rate in chosen Pool III was 2.7% (1/37). If the entire original library of 1201 mutants in fourteen 96-well tissue culture plates had been screened for the BBB transmigration phenotype, we predict that another 25 to 32 mutant candidates in this mutant library with an altered BBB transmigration phenotype would be indentified. Because the putative gene complement of *C. neoformans* is five times greater than the size of the current deletion library, we estimate that there are approximately another 50 to 160 genes that may influence efficient transmigration of *C. neoformans* across the BBB detected in our MBTA system. It is also important to emphasize that none of these single BBB transmigration defective mutants were completely blocked from entering the brain. However, a gene’s presence in a strain might be critically essential during natural infection, as lower yeast numbers in the blood during natural infection likely requires very efficient transmigration mechanisms for CNS entry compared to our high yeast burden MBTA. Another potential limitation of our MBTA model exists in that the single gene deletion mutants were pooled together in our screening experiments or competed directly with the parental strain and strains may have complemented each other. However, we confirmed the novel transmigration affected mutants individually in the HBMEC transcytosis assay, and furthermore examined them in the HBMEC adhesion assay.

High temperature survival (i.e. 37°C) is an important virulence trait in *C. neoformans* [31] and we suspected it had an impact on our MBTA. For instance, mutant strain 4B10 (*nsr1*Δ) showed significantly less yeast cells in the brain than the parental strains CMO18 competition ratio = 0.66±0.04, *P = *0.001) in competitive MBTA. This finding was likely due to growth arrest at 37°C **(** **Figure 3** **)** rather than a specific defect in transmigration. Previously, the gene target deleted in mutant 4B10 (CNAG_02130) was shown to encoded a putative RNA splicing factor homologous to *Neurospora crassa pad1* and was identified as a CSF survival defective mutant [21].

### Invasion Mechanisms

One of the major features in the pathophysiology of cryptococcosis remains its unique neurotropism. The detailed mechanism(s) for this propensity is still uncertain but insights have been gained over the last decade; specifically in the area of transmigration across the BBB. Much of this understanding has been achieved with murine models. First, Chang and Kwon-Chung clearly showed with detailed histopathological studies that *C. neoformans* can migrate transcellularly across the BBB [15]. These findings gave the impetus to Jong and colleagues [18], [20] to identify specific ligands on the yeast and receptors on the host involved in this process. With elegant studies using cryptococcal mutants such as *cps1*Δ, it was shown that the presence of the hyaluronic acid synthase can help binding of yeast to the CD44 receptor of host brain microvascular endothelial cells and transverse these BBB cells through a protein kinase C-controlled system [32]. More recently, it has been documented that a lipid raft-dependent endocytosis process mediates *C. neoformans* internalization into HBMEC and that host CD44 protein, cytoskeleton, and intracellular kinase DYRK3 are involved in this process [33] and hyaluronic acid receptor CD44 deficiency is associated with decreased *C. neoformans* brain infection [34]. These results support the transcellular route as a major mechanism for CNS entry by *C. neoformans*.

*Cryptococcus*, however, is not limited to one mechanism of entry. The ability of urease production to promote microvascular sequestration, enhancing CNS entry has been demonstrated [16]. This paracellular mechanism was further carefully described by Shi and colleagues with the use of real time imaging that demonstrated the importance of this trapping mechanism to allow efficient migration across the intact BBB [6]. A third mechanism for *C. neoformans* entry into the CNS is the classic “Trojan horse” mechanism, where efficiency of transmigration is improved when yeast cells are carried within professional phagocytes [17]. Recently, the production of "titan cells" has been shown to potentially reduce BBB penetration as a result of decreased phagocytosis, and demonstrating importance of yeast cell morphology, for CNS entry with these yeast cells [26].

To evaluate the specific BBB traversal mechanisms in our assay, we performed competition experiments in our MBTA to examine the transcellular route with 12G8 (*cps1*Δ)), paracellular route with 14D6 (*ure1*Δ), and “Trojan horse” route with two novel macrophage adhesion defective mutants. Our model using competition between strains showed that the transcellular route is likely the primary mechanism measured in this specific assay. Therefore, if a mutant is identified as a true CNS transmigration defective yeast in our system, it is more likely due to altered transcytosis rather than use of the other two mechanisms for CNS entry.

### Characteristics of Identified Mutants

Through the MBTA and confirmatory HBMEC transcytosis experiments, two genes in *C. neoformans* were found to be associated with CNS transmigration **(** **Table 3** **)**. Mutant strain 4H12 (*fxn1*Δ) harbors a disruption of gene CNAG_03845 encoding a putative multidrug resistance protein, Fxn1p. In fission yeast, Fxn1 shares sequence similarity to the proton-driven plasma membrane transporters from the multidrug resistance group of the major facilitator superfamily of proteins [35]. *FXN1* plays a role in the entry into G_0_ phase of the cell cycle, possibly by facilitating the release of a signaling substance into the environment as a means of cell-to-cell communication. From a homology search with the *Schizosaccharomyces pombe FXN1* gene, a second gene, *FXN2*, has been identified in *S. pombe* [36]. Green fluorescent protein-fused Fnx1p and Fnx2p localized exclusively to the vacuolar membrane. In both *fnx1Δ* and *fnx2Δ* mutant cells, uptake of lysine, isoleucine, and asparagine were impaired. These results suggest that *fnx1Δ* and *fnx2Δ* are specifically involved in vacuolar amino acid uptake in *S. pombe*. The yeast vacuole functions as both a digestive compartment and also serves as the major storage compartment for basic amino acids in particular. It is not readily apparent from its putative role in *S. pombe* how this gene would be used by *C. neoformans* to affect transmigration of the BBB, but its putative roles as a transporter and for cell-to-cell communications suggest that in *Cryptococcus* this protein facilitates communication between the yeast and host brain endothelial cells during its migration across the host cell. The mutant appeared to also possess a combination of both reduced vascular compartment localization and once in the CNS vascular space poor transmigration across the endothelial membrane. Thus, mechanistically, the mutant may have several reasons for its reduced ability to reach the CNS space. This gene target is now a fertile area to study how it might be used to impact on CNS yeast entry and thus disease progression.

**Table 3:** Characteristics of two novel genes related to CNS transmigration.

Plate_Well	4H12	7F4
**Source**	**Pool III**	**Pool I**
**Gene Name**	***FnxI***	***RubI***
**CNS transmigration**	True disadvantage	True advantage
**Gene Also Known**	*YMR088C02*	*RPS3102*
**Annotation**	MDR Transporter	Ribosomal Chaperone
**Broad Number**	CNAG_03845	CNAG_02827
**Chromosome**	5	3
**Gene Length**	2588 nt	523 nt
**Protein Length**	584 aa	78 aa
**Footnote**	MFS	NEDD8 regulates cullin
**Phnenotype**	Involved in vacuolar amino acid uptake in*S. pombe*; but not known in *C. neoformans*	“Slow grower”; “petite” cells; CNS survival defect

Abbreviation: Fnx: facilitated nutritional exit; Rub: related to ubiqutin; MDR: multidrug resistance; MFS: major facilitator superfamily; NEDD8: neural precursor cell-expressed developmentally down-reb gulated gene 8; nt: nucleotide; aa: amino acid; HBMEC: human brain microvascular endothelial cell.

Interestingly, we initially detected mutant strain 7F4 due to its absence in the CNS from the pooled inocula in the screening MBTA. In contrast, when directly compared to the parental strain it more efficiently traversed the BBB in the MBTA. We speculate that the absence of the mutant 7F4 from the CNS in our screening MBTA was due to its slow growth at higher temperatures in both the pooled inoculum and the mouse brain **(** **Figure 3** **)**. For instance, we did not prolong growth time for 7F4 during preparation of pooled inoculum and also during the harvest of yeast cells recovered from brain homogenate for PCR. In contrast, in the competitive MBTA with two strains we quantified equal numbers of 7F4 and CMO18 in inoculum and we extended growth time to 4 days. We hypothesize that the small yeast size of the “petite” mutant **(** **Figure 6A and B** **)** of 7F4 facilitates its physical crossing through HBMEC [26]. The gene deleted in mutant strain 7F4 (CNAG_02827) encodes a putative ribosomal chaperone with homology to *RUB1* in *S. cerevisiae*. It is an ubiquitin-like protein that post-translationally neddylates cullin proteins and binds to cullins, notably Cdc53p in *S. cerevisiae* [**37**]. Defects in the neddylation pathway in yeasts result in severe defects in cell growth and development. On the other hand, despite its efficient movement across BBB, 7F4 has a hypovirulence phenotype due to its decreased survival in the CNS [21].

In summary, we describe a MBTA to elucidate the genetic controls influencing the movement of *Cryptococcus* across the BBB. Specifically, we identify two new genes (*FNX1* and *RUB1*) related to efficient cryptococcal BBB transmigration. Further exploration of the genetic and molecular mechanism(s) for cryptococcal neurotropism using our MBTA may lead to new understandings of how this fungus uniquely crosses the BBB. With this knowledge, we may be able to predict and/or develop strategies or drugs to interrupt this yeast’s ability to enter this critical site of infection. Alternatively, we may be to predict from yeast genome studies which strains isolated from other body sites such as the lung are most likely to efficiently cross the BBB and cause life-threatening disease.

## Materials and Methods

### Cryptococcal Mutants

We obtained a gene deletion library from the Fungal Genetic Stock Center (FGSC; www.fgsc.net) [38] that included 1201 signature-tagged, targeted gene deletion strains of *C. neoformans* var. *grubii* using a NAT^R^ cassette [12], [39]. The parental strain for the mutant library, CMO18, is a passaged isolate of *C. neoformans* strain H99.

### MBTA as an Experimental Meningoencephalitis Model

In order to focus on cryptococcus in its traversal across the BBB, we utilized the MBTA modified from a tail vein infection model [24] in order to bypass the lung entry route. Briefly, BALB/c female mice (8–10 week-old; body weight between 15–25 g; Charles River Laboratory US and Biolasco Taiwan) were housed at the Duke University Medical Center vivarium and Mackay Memorial Hospital vivarium and given food and water ad libitum. The MBTA protocol was approved by the Duke Institutional Animal Care and Use Committee and Mackay Memorial Hospital Animal Care and Use Committee. Mouse tails were initially heated to increase blood flow in the tail vein. The mice were then transferred to a restraint that allows access to the tail vein using a 27g needle. Each mouse was injected with 0.1–0.2 ml of the inoculum. After 24 hrs post-infection, mice were euthanized by CO_2_ asphyxiation. A blood sample (0.3 to 0.6 ml) was withdrawn by cardiac puncture of the right ventricle using an EDTA pre-rinsed syringe and then collected into an EDTA pre-rinsed Eppendorf tube. To avoid contamination of brain tissue by circulating cryptococcal yeast cells, an entire circulation perfusion was then performed by injecting sterile saline (30–50 ml) into the left ventricle until whitening of the organs, with the right and left atrium and inferior vena cava being cut open to allow drainage during the procedure [7]. The brain was then removed, weighed, and homogenized in 2 ml of sterile PBS. The homogenates were grown on chloramphenicol-containing plates. Appropriate dilutions of the homogenates were plated (100 µl each) in triplicate onto SDA/c plates and CFUs were enumerated after 48 hrs of growth at 30°C. The yeast colonies from mouse brain homogenates were used for either preparation of genomic DNA for the screening MBTA or for determination of a competition ratio of mutant (NAT-resistant) to parent (NAT susceptible) in the competitive MBTA. Three to five mice were harvested per group for statistical comparisons.

### Screening MBTA

Prior to use in the mouse studies, yeast strains were replica plated from −80°C stocks of mutant pools and were grown individually in YPD broth (1% yeast extract, 2% peptone, 2% dextrose) in 96-well deep-well blocks at 30°C incubation without shaking for 3 days. For the purpose of the screen, equal volumes (200 µl) of yeasts per mutant from 48 wells were harvested, pooled, washed three times with sterile phosphate buffered saline (PBS). After pooling, the number of viable yeast cells for each pool was quantified using trypan blue dye exclusion and the concentration confirmed by hemacytometer count. After preparation of the pooled mutants, the MBTA was applied to screen the pooled mutants. *C. neoformans* genomic DNA from the yeast colonies of mouse brain homogenates was prepared using cetyltrimethylammonium bromide (CTAB) followed by phenol-chloroform extraction as previously described [40], [41] for large amounts of DNA for signature tag sequence PCR identification. For signature tag sequence reconfirmation, genomic DNA was isolated from *Cryptococcus* strains using a previously described miniprep technique [42]. We used the 48 tag-specific amplification primers and a common amplification primer found in the NAT deletion cassette (5′-GCATGCCCTGCCCCTAAGAATTCG-3′) to amplify the signature tags as described elsewhere [12].

### Competition MBTA

Strains were grown individually on YPD medium in conical tubes at 30°C incubation with shaking for 18 to 20 hrs. We quantified equal numbers of viable yeast cells from both an individual mutant and the CMO18 parental strain using trypan blue dye exclusion and adjusted the concentration by hemacytometer. The strains were pre-mixed in a 1∶1 ratio directly before animal inoculations were performed. For example, the inoculum of 10^7^ CFU of yeast cells was prepared as a 1∶1 ratio combination of 5×10^6^ CFU of individual mutant and 5×10^6^ CFU of CMO18. The “Adjustment Factor” (AF) is defined as a CFU ratio of individual mutant to parental strain CMO18 in the pre-mixed inoculum. Ideally, the AF was 1.0. An aliquot of either the premixed inoculum or harvested brain homogenates was serially diluted, and an appropriate volume of the dilution was plated in triplicate on SDA/c plates to get 100–200 CFUs per plate. All of the colonies grown on SDA/c plates were then transferred individually to SDA/c NAT containing plates. The ratio of the CFU of mutant strain colonies (NAT resistant) to parental strain colonies (NAT susceptible) was defined as the competition ratio.

### Entrapment MBTA

Entrapment MBAT was done through tail vein injection of single candidate mutant. After 24 hours, mice were anesthetized using Zoletil 50 (Tiletamine and Zolazepam) (0.5 ml/25 mg/kg) intramuscular (IM) injection plus Xylazine (0.5 ml/10 mg/kg) and then transcardially perfused with 50 ml of PBS followed by 50 ml of 4% paraformaldehyde. Perfused brains were fixed in 4% paraformaldehyde overnight in 4°C, and paraffin-embedded. The brains were then cut into 5-µm coronal sections, mounted on the slides and processed for staining. At least five coronal sections at the same brain location for each mouse were examined and all the yeast cells on each section were counted. Mucin staining was used to help identify the different brain regions and identify specific yeast cells location. For quantification purposes, the number of mucin-positive cells per high-power field was assessed by a single observer. Entrapped percentage (%) is defined as percentage of intra-microvascular cryptococci (entrapped in microvessels) divided by total yeast cells in the brain (×100%). The ratio of entrapped percentage is defined as a ratio of candidate mutant to parental strain in entrapped percentage. Each experiment was performed three times.

### Growth Curve

*Cryptococcus* yeast cells grew in 10 ml YPD broth in conical tubes at 30°C shaking incubator for 48 hrs. Yeast cells were washed with PBS three times, adjusted to 10^6 ^CFU/ml in 60 ml YPD broth, and incubated at both 30°C and 37°C in shake culture for four days. In order to confirm inoculum concentrations, we plated 0.1 ml of a 10^−3^ serial dilution of an original cell suspension on SDA/c plates to obtain approximately 100 CFUs per plate. OD 600 nm absorbance at both 30°C and 37°C were measured over 92 hrs at set time-points: 0, 4, 20, 24, 28, 44, 48, 52, 69, 72, 76 and 92 hr.

### HBMEC Transcytosis Assay

*C. neoformans in vitro* transcytosis assay was performed as described [14]. Briefly, 10^3^ HBMEC were cultured on collagen-coated Transwell polycarbonate tissue-culture inserts with a pore diameter of 8 µm (Corning Inc., Corning, NY). Integrity of the monolayer was confirmed by measurement of the trans-endothelial electrical resistance (TEER), which was 250∼300 Ω per cm^2^, as measured with an Endohm volt/ohm meter (World Precision Instruments) for 4–5 days. To measure transcytosis, HBMEC monolayers were washed with experimental medium and 10^6^ yeast cells were added to the upper chamber (total volume 200 µl). The cultures were incubated at 37°C with 5% CO_2_. At 1, 3, and 6 hr post-inoculation, the top transwells were transferred to a new culture plate with fresh medium on the bottom. Cells were counted at each timepoint for the spent medium; transcytosis was calculated as the sum of all three timepoints. For example, if we count 100 cells at 1 hr and 120 cells at 3 hr, then at the 3 hr timepoint the total transcytosed yeast cells are 100+120 = 220. The spent medium from each well was fixed with 3.7% formaldehyde prior to counting. Then samples were collected by centrifugation, and *C. neoformans* cells were directly counted using a hemacytometer. Triplicate samples were measured for each strain and the experiment was repeated 3 times. The integrity of the HBMEC monolayer was constantly assessed by both measurement of TEER and assessing movement of horse radish peroxidase activity from upper to lower chambers. Under these conditions, TEER was maintained and no movement of horseradish peroxidase activity was detected at 16 hr.

### HBMEC Adhesion Assay

The *in vitro* binding assay of *C. neoformans* to HBMEC was performed as previously described [14], [20], [43]. Briefly, HBMEC were grown to confluence in collagen-coated 24-well tissue culture plates (Corning Inc., Corning, NY). 10^6^ yeast cells were suspended in 0.5 ml of medium and added to the HBMEC monolayer at a multiplicity of infection (MOI) of 10 and incubated for 2 h at 37°C. Unattached yeast cells were then removed by washing with medium four times. HBMEC were then lysed with 0.5% TritonX-100, diluted, and plated onto blood or YPD agar plates to determine the CFU associated with HBMEC. Triplicate cultures were measured for each strain and the experiment was repeated 3 times. Results of adhesion percentage are presented as the percent (%) adhesion of inocula: [(number of CFU recovered)/(number of CFU inoculated)] ×100%. The ratio of adhesion percentage is defined as a ratio of candidate mutant to parental strain in adhesion percentage.

### Measurement of *Cryptococcus* Colony and Yeast Cell Size

Yeast cell measurements were performed on candidate mutants by India ink staining as previously described [44], with the following modifications. Cells were examined under oil immersion at 63X magnification following 48 hrs incubation in Eagle's Media at 30°C with shaking. Cell diameter was measured using Adobe Photoshop (Adobe Inc., San Jose, CA), with a total of 100 cells measured per strain.

### Screening MMPA

We used an abbreviated MMPA [45] to screen for cryptococcal mutants that had adhesion/survival defects to professional phagocytes. Briefly, J774A.1 cells (a murine BALB/c macrophage-like cell line derived from a reticulum cell sarcoma) were harvested, centrifuged, resuspended, and counted by hemacytometer in the presence of trypan blue to assess viability; 8×10^5^ J774A.1 cells were added per well in DMEM (Dulbecco's Modified Eagle Medium; Life Technologies, Grand Island, NY) containing gamma-interferon (100 U/ml) and lipopolysacharide (0.6 µg/ml) well for macrophage activation and incubated at 37°C with 5% CO_2_ for 12–18 hrs.

“Inoculated Pool (I)” of cryptococcal mutants was prepared as previously described above. Following the final wash with PBS, the yeast cells were resuspended in 1 ml of DMEM and incubated for opsonization with monoclonal antibody (mAB18B7) (1 µg/ml) (a kind gift from Arturo Casadevall, Albert Einstein College of Medicine, Bronx, New York) [46] for one hour at 37°C with 5% CO_2._ Old tissue culture medium from activated macrophage wells was removed, and gently replaced with the opsonized 4×10^6^ yeast cells in DMEM to each macrophage well. Opsonized 4×10^6^ cryptococcal cells and activated 8×10^5^ macrophage cells were co-cultured in a tissue culture incubator for 0.5 to 1 hour. “Wash Pool (W)” was obtained by removing spent medium and washing each well gently 3 times with PBS to remove extracellular yeasts. After adding new tissue culture medium, each well was incubated another 24 hrs and then “Lysate Pool (L)” in each well was acquired with an addition of a 0.5% sodium dodecyl sulfate (SDS) solution. Appropriate dilutions were plated on SDA/c plates and these plates were incubated at 30°C for 48–72 hrs to quantify phagocyte killing by CFU counts.

Fungal genomic DNA of I, W and L Pools were extracted using the “smash and grab” miniprep protocol described above. Negative selection for macrophage adhesion/survival defective mutants was determined by identifying loss of signature tag PCR bands in “L” Pools but present in either “W” Pools or “I” Pools.

### Identification of Macrophage Adhesion Defective Mutants

After the screen in the MMPA with yeast cell pools, individual phagocytosis/survival mutants selected from this initial MMPA screen were grown on YPD medium at 30°C overnight. The number of candidate mutants and CMO18 were quantified by hemacytometer using trypan blue dye exclusion and concentrations were adjusted to numbers of macrophages (see MMPA). “I” pools and “W” pools were collected by after 1 hour incubation and the resulting adhesion capability for each pool was expressed as the percent (%) adhesion of inocula: [(number of CFU recovered)/(number of CFU inoculum)] ×100%, that is [(I–W)/(I) ] ×100%. Three measurements were made for each sample. CMO18 was used as reference strain. All adhesion experiments were repeated two times.

### Statistics

All data were analyzed using Microsoft Excel (Microsoft Inc., Redmond, WA) unless otherwise stated. Data were expressed as mean ± SD of at least three repeat experiments. All experiments were performed in triplicate. Student’s t test was used to determine the statistical significance between the parental strain control and mutant treatment groups. *P* value <0.05 was considered statistically significant. Asterisk (*) means *P*<0.05, (**) means *P*<0.01, and (***) means *P*<0.001.
